# Supplementary material for: The Effect of Butyrate-Supplemented Parenteral Nutrition on Intestinal Defence Mechanisms and the Parenteral Nutrition-Induced Shift in the Gut Microbiota in the Rat Model
Source: Biomed Res Int. 2019 Feb 28;2019:7084734. doi: 10.1155/2019/7084734 (PMC6421034; doi:10.1155/2019/7084734)
Supplement: Supplementary Materials — Table S1: composition of parenteral nutrition mixture. Table S2: statistical analysis of sequencing data. Figure S1: stability of the antigens of interest after one freezing/thawing cycle. [file 7084734.f1.pdf]

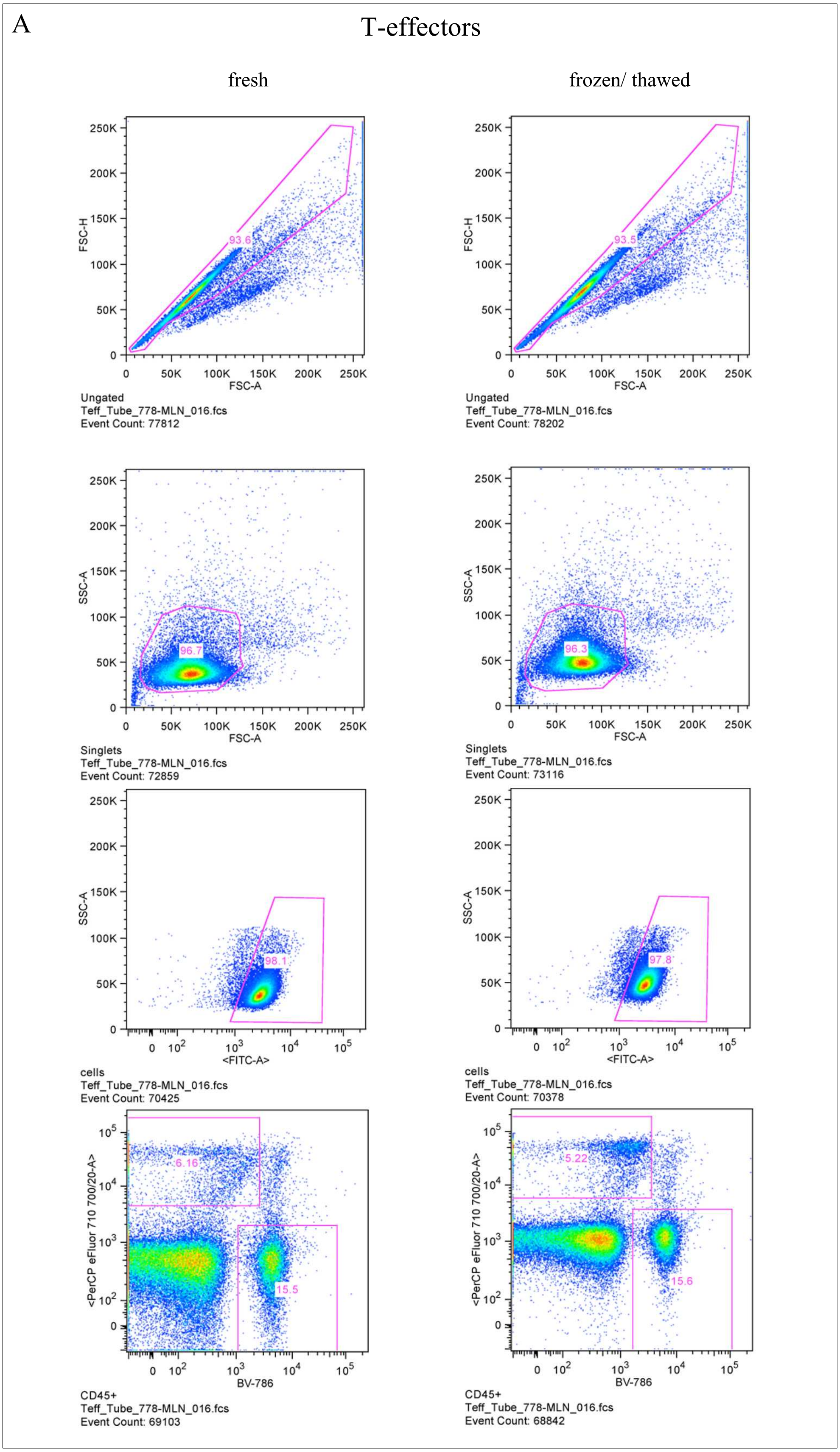
A
T-effectors
fresh
frozen/ thawed
FSC-H
FSC-A
93.6
93.5
Ungated
Teff_Tube_778-MLN_016.fcs
Event Count: 77812
Event Count: 78202
SSC-A
96.7
96.3
Singlets
Event Count: 72859
Event Count: 73116
<FITC-A>
98.1
97.8
cells
Event Count: 70425
Event Count: 70378
<PerCP eFluor 710 700/20-A>
BV-786
6.16
15.5
5.22
15.6
CD45+
Event Count: 69103
Event Count: 68842

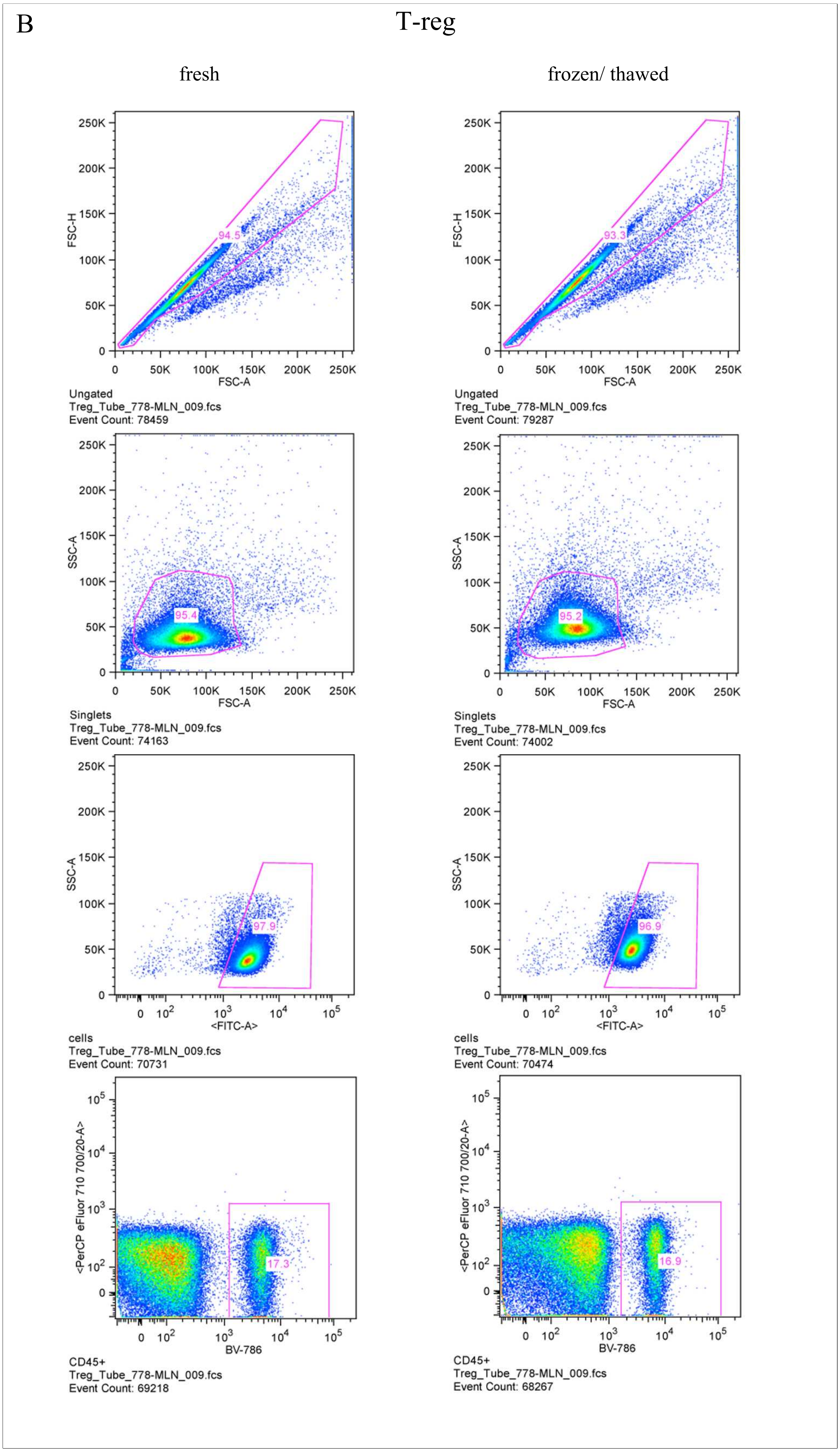

Figure S1: The effect of the one freezing/ thawing cycle on the lymfocyte antigens expression in effector (A) and regulatory (B) T cells.

S1 Table Composition of parenteral nutrition mixture

Ingredients	kcal . kg^{-1} . day^{-1}	% of non-amino acid energy
amino acid solution (Aminoplasmal 15%)	5	
glucose	140	70
lipid emulsion (Intralipid 20%)	60	30
total energy	205	

Crude elements

Amino acid composition

	mg . kg^{-1} . day^{-1}
Isoleucine	47
Leucine	91
Lysine	64
Methionine	46
Phenylalanine	46
Threonine	43
Tryptophan	17
Valine	58
Arginine	128
Histidine	42
Glycine	154
Alanine	179
Proline	59
Aspartic acid	64
Cysteine	3
Glutamic acid	130
Serine	24
Tyrosine	4

Vitamins

	µg . kg^{-1} . day^{-1}
Thiamin (B_1)	124
Riboflavin B_2)	140
Pyridoxine (B_6)	155
Cyancobalamine (B_{12})	0.2
Nicotinamide	1552
Panthothenic acid	582
Ascorbic acid (C)	3880
Biotin	2.3
Folic acid	15.5

Electrolytes

	mmol . kg^{-1} . day^{-1}
Na^+	15.6
K^+	1.05
Mg^{2+}	0.45
Ca^{2+}	0.18
Cl^-	16.1
SO_4^{2-}	0.45
HPO_4^{2-}	0.21
H_2PO^{4-}	0.11

Trace elements

	µmol . kg^{-1} . day^{-1}
Cr^{3+}	0.008
Cu^{2+}	0.233
Fe^{3+}	0.776
Mn^{2+}	0.039
Mo^{6+}	0.008
Se^{4+}	0.039
Zn^{2+}	2.988
I^-	0.039
F^-	1.940

Supplementary table S2 Statistical analysis of sequencing data: genus level

Bacteroidetes;D_2__Bacteroidia;D_3__Bacteroidales;D_4__Bacteroidaceae;D_5__Bacteroides
Bacteroidetes;D_2__Bacteroidia;D_3__Bacteroidales;D_4__Bacteroidales S24-7 uncultured bacterium
Bacteroidetes;D_2__Bacteroidia;D_3__Bacteroidales;D_4__Porphyromonadaceae;D_5__Parabacteroides
Bacteroidetes;D_2__Bacteroidia;D_3__Bacteroidales;D_4__Prevotellaceae;D_5__Alloprevotella
Bacteroidetes;D_2__Bacteroidia;D_3__Bacteroidales;D_4__Prevotellaceae;D_5__Prevotellaceae Ga6A1 group
Bacteroidetes;D_2__Bacteroidia;D_3__Bacteroidales;D_4__Prevotellaceae;D_5__Prevotellaceae NK3B31
Bacteroidetes;D_2__Bacteroidia;D_3__Bacteroidales;D_4__Prevotellaceae;D_5__Prevotellaceae UCG-001
Bacteroidetes;D_2__Bacteroidia;D_3__Bacteroidales;D_4__Rikenellaceae;D_5__Alistipes
Bacteroidetes;D_2__Bacteroidia;D_3__Bacteroidales;D_4__Rikenellaceae;D_5__Rikenellaceae RC9 gut group
Cyanobacteria;D_2__Melainabacteria;D_3__Gastranaerophilales;D_4__uncultured bacterium
D_1__Firmicutes;D_2__Bacilli;D_3__Lactobacillales;D_4__Lactobacillaceae;D_5__Lactobacillus
Firmicutes;D_2__Clostridia;D_3__Clostridiales;D_4__Christensenellaceae;D_5__Christensenellaceae R-7 group
Firmicutes;D_2__Clostridia;D_3__Clostridiales;D_4__Lachnospiraceae;D_5__Anaerostipes
Firmicutes;D_2__Clostridia;D_3__Clostridiales;D_4__Lachnospiraceae;D_5__Blautia
Firmicutes;D_2__Clostridia;D_3__Clostridiales;D_4__Lachnospiraceae;D_5__Coprococcus
Firmicutes;D_2__Clostridia;D_3__Clostridiales;D_4__Lachnospiraceae;D_5__Coprococcus
Firmicutes;D_2__Clostridia;D_3__Clostridiales;D_4__Lachnospiraceae;D_5__Incertae sedis
Firmicutes;D_2__Clostridia;D_3__Clostridiales;D_4__Lachnospiraceae;D_5__Lachnoclostridium
Firmicutes;D_2__Clostridia;D_3__Clostridiales;D_4__Lachnospiraceae;D_5__Lachnospiraceae AC2044
Firmicutes;D_2__Clostridia;D_3__Clostridiales;D_4__Lachnospiraceae;D_5__Lachnospiraceae NC2004
Firmicutes;D_2__Clostridia;D_3__Clostridiales;D_4__Lachnospiraceae;D_5__Lachnospiraceae NK4A136
Firmicutes;D_2__Clostridia;D_3__Clostridiales;D_4__Lachnospiraceae;D_5__Lachnospiraceae UCG-001
Firmicutes;D_2__Clostridia;D_3__Clostridiales;D_4__Lachnospiraceae;D_5__Roseburia
Firmicutes;D_2__Clostridia;D_3__Clostridiales;D_4__Lachnospiraceae;D_5__uncultured
Firmicutes;D_2__Clostridia;D_3__Clostridiales;D_4__Lachnospiraceae;Other
Firmicutes;D_2__Clostridia;D_3__Clostridiales;D_4__Peptococcaceae;D_5__uncultured
Firmicutes;D_2__Clostridia;D_3__Clostridiales;D_4__Peptostreptococcaceae;D_5__Peptoclostridium
Firmicutes;D_2__Clostridia;D_3__Clostridiales;D_4__Ruminococcaceae;D_5__Oscillibacter
Firmicutes;D_2__Clostridia;D_3__Clostridiales;D_4__Ruminococcaceae;D_5__Ruminiclostridium
Firmicutes;D_2__Clostridia;D_3__Clostridiales;D_4__Ruminococcaceae;D_5__Ruminococcaceae NK4A214
Firmicutes;D_2__Clostridia;D_3__Clostridiales;D_4__Ruminococcaceae;D_5__Ruminococcaceae UCG-005
Firmicutes;D_2__Clostridia;D_3__Clostridiales;D_4__Ruminococcaceae;D_5__Ruminococcaceae UCG-014
Firmicutes;D_2__Clostridia;D_3__Clostridiales;D_4__Ruminococcaceae;D_5__Ruminococcus
Firmicutes;D_2__Clostridia;D_3__Clostridiales;D_4__Ruminococcaceae;D_5__[Eubacterium] coprostan group
Firmicutes;D_2__Clostridia;D_3__Clostridiales;D_4__Ruminococcaceae;D_5__uncultured
Firmicutes;D_2__Erysipelotrichia;D_3__Erysipelotrichales;D_4__Erysipelotrichaceae;D_5__Allobaculum
Firmicutes;D_2__Erysipelotrichia;D_3__Erysipelotrichales;D_4__Erysipelotrichaceae;D_5__Candidatus Stoquefich
Firmicutes;D_2__Erysipelotrichia;D_3__Erysipelotrichales;D_4__Erysipelotrichaceae;D_5__Turicibacter
Firmicutes;D_2__Erysipelotrichia;D_3__Erysipelotrichales;D_4__Erysipelotrichaceae;Other
Proteobacteria;D_2__Alphaproteobacteria;D_3__Rhodospirillales;D_4__Rhodospirillaceae;D_5__Thalassospira

Proteobacteria;D_2__Betaproteobacteria;D_3__Burkholderiales;D_4__Alcaligenaceae;D_5__Parasutterella
Proteobacteria;D_2__Deltaproteobacteria;D_3__Desulfovibrionales;D_4__Desulfovibrionaceae;D_5__Desulfovib
Proteobacteria;D_2__Gammaproteobacteria;D_3__Enterobacteriales;D_4__Enterobacteriaceae;D_5__Escherichi
Proteobacteria;D_2__Gammaproteobacteria;D_3__Enterobacteriales;D_4__Enterobacteriaceae;Other
Verrucomicrobia;D_2__Verrucomicrobiae;D_3__Verrucomicrobiales;D_4__Verrucomicrobiaceae;D_5__Akkerman
Unassigned;Other;Other;Other;Other;Other

Fitted ANOVA model					Tukey's HSD pairwise comparis			
Coefficients					Effect size: fold change (95% CI)			
Intercept (control)	PN+But	PN	p-value	adjusted p-value	PN+But to control	PN to control	PN+But to PN	PN+But to control
-0.214	4.574	2.944	0.001	0.010	4.574	2.944	-1.629	0.001
3.842	-1.003	-0.884	0.222	0.387	-1.003	-0.884	0.119	0.257
-2.571	3.730	3.973	0.000	0.005	3.730	3.973	0.243	0.000
-1.459	0.945	-0.992	0.568	0.653	0.945	-0.992	-1.937	0.846
2.814	-3.390	-0.869	0.004	0.029	-3.390	-0.869	2.521	0.003
2.203	-2.312	-2.944	0.002	0.020	-2.312	-2.944	-0.632	0.014
2.105	-0.146	-0.979	0.579	0.653	-0.146	-0.979	-0.832	0.988
-0.371	2.164	1.679	0.008	0.046	2.164	1.679	-0.485	0.009
-0.731	0.731	0.727	0.681	0.727	0.731	0.727	-0.004	0.732
0.840	-1.486	1.607	0.015	0.069	-1.486	1.607	3.093	0.231
0.648	-3.128	-2.088	0.028	0.082	-3.128	-2.088	1.041	0.026
-1.203	0.847	0.135	0.485	0.633	0.847	0.135	-0.712	0.488
0.148	-1.658	-4.118	0.001	0.008	-1.658	-4.118	-2.459	0.118
0.182	1.194	1.214	0.280	0.425	1.194	1.214	0.020	0.361
-0.713	-0.212	0.486	0.562	0.653	-0.212	0.486	0.699	0.939
-1.083	1.083	0.217	0.597	0.653	1.083	0.217	-0.866	0.592
0.221	-1.398	-1.591	0.338	0.497	-1.398	-1.591	-0.193	0.463
0.016	0.326	-1.068	0.087	0.194	0.326	-1.068	-1.394	0.841
-1.212	1.445	-0.785	0.038	0.099	1.445	-0.785	-2.230	0.166
0.039	-1.342	-1.252	0.370	0.499	-1.342	-1.252	0.091	0.425
3.480	-2.164	-2.043	0.055	0.136	-2.164	-2.043	0.121	0.081
0.135	-1.665	-1.872	0.130	0.266	-1.665	-1.872	-0.207	0.225
0.278	0.919	-0.737	0.078	0.183	0.919	-0.737	-1.657	0.348
2.377	-1.190	-0.753	0.243	0.394	-1.190	-0.753	0.437	0.227
0.364	-1.417	-1.075	0.165	0.310	-1.417	-1.075	0.342	0.170
-0.377	1.273	0.087	0.110	0.235	1.273	0.087	-1.186	0.128
0.238	-0.252	0.413	0.573	0.653	-0.252	0.413	0.665	0.907
0.095	-0.765	-0.057	0.366	0.499	-0.765	-0.057	0.708	0.391
1.251	-0.287	0.068	0.862	0.881	-0.287	0.068	0.355	0.903
-1.050	0.748	1.381	0.014	0.069	0.748	1.381	0.632	0.185
-0.340	-0.184	1.353	0.007	0.044	-0.184	1.353	1.537	0.899
0.240	-0.473	0.076	0.553	0.653	-0.473	0.076	0.549	0.640
0.801	-2.283	-1.355	0.021	0.081	-2.283	-1.355	0.929	0.017
-0.665	1.018	1.300	0.028	0.082	1.018	1.300	0.282	0.096
1.395	-0.142	0.662	0.372	0.499	-0.142	0.662	0.804	0.966
-1.543	1.232	2.809	0.030	0.082	1.232	2.809	1.577	0.399
-1.749	0.686	-1.816	0.233	0.392	0.686	-1.816	-2.502	0.873
-0.897	-1.089	0.333	0.274	0.425	-1.089	0.333	1.422	0.427
-0.789	-0.556	-1.730	0.202	0.365	-0.556	-1.730	-1.174	0.820
-1.498	0.634	2.258	0.138	0.270	0.634	2.258	1.624	0.826

-1.135	2.046	2.618	0.000	0.005	2.046	2.618	0.572	0.002
-0.825	0.373	3.375	0.021	0.081	0.373	3.375	3.002	0.940
-2.896	3.979	3.590	0.024	0.082	3.979	3.590	-0.389	0.035
-0.716	-0.609	-1.496	0.591	0.653	-0.609	-1.496	-0.886	0.906
1.648	-1.717	-3.521	0.026	0.082	-1.717	-3.521	1.804	0.313
0.200	0.320	0.420	0.724	0.756	0.320	0.420	0.100	0.830

son	
p-values	
PN to control	PN+But to PN
0.019	0.250
0.340	0.981
0.000	0.944
0.832	0.538
0.558	0.030
0.003	0.666
0.584	0.696
0.039	0.729
0.735	1.000
0.186	0.011
0.150	0.615
0.981	0.622
0.000	0.023
0.349	1.000
0.724	0.550
0.978	0.732
0.376	0.986
0.191	0.089
0.557	0.033
0.472	0.996
0.102	0.991
0.160	0.977
0.496	0.065
0.529	0.816
0.339	0.897
0.989	0.185
0.772	0.550
0.994	0.473
0.994	0.867
0.011	0.316
0.016	0.010
0.988	0.579
0.176	0.445
0.031	0.821
0.491	0.389
0.023	0.264
0.410	0.228
0.919	0.277
0.182	0.460
0.126	0.343

0.000	0.503
0.024	0.052
0.058	0.962
0.563	0.826
0.021	0.062
0.728	0.983
